# Piles and Prolapsus Ani*Read at a meeting of the Middlesex-East Medical Society, February 25, 1885.

**Published:** 1885-06-20

**Authors:** W. Symington Brown

**Affiliations:** Stoneham, Mass.


### PILES AND PROLAPSUS ANI.*

By W. SYMINGTON BROWN, M. D., Stoneham, Mass.

No surgical disease that I know of is so common as piles; few can be as certainly cured; and yet it seems to me that the radical remedy, removal by the ecraseur, is seldom resorted to by general practitioners. I have several times drawn the attention of this Society to the importance of dilating the sphincter ani before

*Read at a meeting of the Middlesex-East Medical Society, February 25, 1885.

operating for hemorrhoids. The late Dr. Van Buren, of New York, was the inventor of this method, and Dr. H. R. Storer brought it prominently before the profession. Both thumbs are inserted into the anus (while the patient is anesthetized until it is paralyzed). A sound like tearing may be heard, but the muscle is not torn, only paralyzed. This preliminary operation is essential; it allows us to examine the parts thoroughly. In women, with two fingers in the vagina, the bowel can be readily turned inside out. In men, we may insert Sims's speculum. If hemorrhage should occur during the operation we can more easily control it, because there is ample room to work in.

I have always employed the wire ecraseur, never the ligature, to remove piles, and since 1865 have had twenty-six cases. I do not say that the ligature should never be used. Surgeons with great experience advise it, but the application of Sir Spencer Wells's forceps, hot water torsion, and working the ecraseur slowly, have enabled me to dispense with ligatures so far.

Last October I attended a male tramp at our Poor Farm, Stoneham, for prolapsus ani. The protrusion was nearly as large as my fist, and, according to the man's own statement, had been down for several days. He had also retention of urine. After emptying the bladder with a soft rubber catheter, and instructing him how to use the instrument, I placed him in the knee elbow position, washed the protruded gut with warm water, painted the whole surface with a dilute solution of iodine, smeared the parts with vaseline, and without much effort succeeded in returning the mass.

After it had been cleansed I made a careful examination to find out the nature of the prolapse. Prof. Van Buren was the first in this country to point out that the accident has four varieties, namely, (1) partial (only mucous membrane); (2) complete (all the coats of the rectum); (3) invaginated (rectum telescoped appears externally); (4) high invagination.

A partial prolapse, involving only the mucous membrane, is a comparatively simple trouble and occurs most frequently in delicate, badly nourished children. The complete variety may be distinguished by transverse deep rugæ. "The greased finger, passed around the base of the tumor, recognizes that its external surface is absolutely continuous with the membrane that lines the orifice of the anus. In the third variety the finger can be inserted into a groove alongside of the base of the tumor, so as to recognize a distinct sulcus." This was the kind I had to deal with. After the mass had been returned I found that the patient had marked enlargement of the prostrate gland, the original source of the trouble in this case; for difficulty in passing water necessitated straining, and that had brought down a large portion of the rectum.

A few general remarks on this affection may not be out of place. We meet it more frequently among children than adults. In the former it is generally produced by constipation or diarrhœa, and sometimes results from the child being allowed to sit too long at a time on the chamber-pot. Ascarides also occasionally produce so much irritation in the neighborhood of the anus that the bowel, or a portion of its mucous membrane, comes down. The presence of a polypus may also cause it.

In recent cases the protrusion is readily returned by partially inverting the child, applying a large sponge dipped in hot water for a few minutes, greasing the parts with lard or vaseline, and applying gentle, steady pressure with the fingers. In scared children it may be necessary to administer ether. In sickly, scrofulous patients the prolapse is apt to recur again and again, and the sphincter ani becomes not only relaxed but atrophied. In such cases hot-water injections containing a decoction of oak-bark prove serviceable, and it is a good plan for the mother or nurse to support the parts during defecation. If the anus is drawn aside by a broad strip of sticking plaster applied to one buttock, the tendency to prolapse is much lessened.

In one case, an adult, I was obliged to insert a piece of candle as an internal support. The candle was passed entirely within the sphincter, with a loop of tape attached to the wick. In my last case, already referred to, a compress and T bandage kept up the bowel. Straining was avoided by regularly emptying the bladder with a catheter.

If milder measures fail, the sure remedy is a simple surgical operation. A copious cleansing injection is first administered, then a Sims's speculum (preferably made of wood or horn*) is inserted into the anus, and the mucous membrane dried with Japanese bibulous paper, such as is used by dentists. The actual cautery is then applied in several straight lines according to the amount of contraction needed. Three or four lines are generally sufficient. The iron should be at a dull-red heat. Paquelin's thermo-cautery answers the purpose very well. The subacute inflammation thus set up glues the tissues together and prevents a recurrence of the prolapse.

The high invaginated variety is not as the other three ; but is more likely to be mistaken for a rectal polypus; a mistake which has happened more than once, followed by disastrous results. Sometimes the whole of the rectum and a portion of the sigmoid flexure come down, and one or two cases are on record where a portion of the ileum has passed through the ileo-cecal valve, and the invagination has proceeded until the gut appeared externally. There is always a narrow passage left through which liquid feces may be discharged. Where the diagnosis is clear, and has been made out early, abdominal section for the purpose of disentangling the bowel is the only sure remedy. In all operations on a prolapsed rectum it is necessary to recollect that the peritoneum descends much lower anteriorly than posteriorly, and that it is only the terminal inch of the rectum which has no serous covering. In cases of complete prolapsus ani of long standing, if we attempted to remove the mass we would probably open the peritoneal cavity, and light up fatal peritonitis.—*Louisville Med. Jour.*

---

*One made of wood was exhibited.

